# The Batrachian Barf Bowl: An authentic research experience using ecological data from frog diets

**DOI:** 10.1002/ece3.9095

**Published:** 2022-07-17

**Authors:** Joanna G. Larson, Hayley L. Crowell, Lisa L. Walsh, Alison R. Davis Rabosky

**Affiliations:** ^1^ Department of Ecology and Evolutionary Biology and Museum of Zoology University of Michigan Ann Arbor Michigan USA; ^2^ Department of Biological Sciences University of Notre Dame Notre Dame Indiana USA; ^3^ Education Research & Outreach Donald Danforth Plant Science Center St. Louis Missouri USA

**Keywords:** authentic research experience (ARE), biodiversity, collaboration, education, frog, herpetology, inter‐institutional, museum collections, remote learning, undergraduate

## Abstract

Authentic research experiences (AREs) are a powerful strategy for inspiring and retaining students in science, technology, engineering, and math (STEM) fields. However, recent demand for virtual learning has emphasized the need for remote AREs that also foster a sense of community and interpersonal connections among participants. Here, we describe an ARE activity that leverages digitized diet data from natural history collections to provide students with collaborative research experience across any learning environment. Using magnified photographs of frog stomach contents collected in the Peruvian Amazon, we designed an open‐source “bowl game” competition that challenges students to identify, measure, and compare diet items across vouchered frog specimens (“Batrachian Barf Bowl”). To demonstrate learning outcomes, we ran this activity with 39 herpetology class students from the University of Notre Dame and the University of Michigan. We used pre‐ and post‐activity assessments to evaluate effectiveness, scientific accuracy of results, and impact on student well‐being. With minimal preparation and training in invertebrate identification, students were successful in identifying hundreds of frog diet items to taxonomic order, although accuracy varied among clades (global accuracy ~70%). While we found no difference in science identity, community, or self‐efficacy between the two institutions at either time point (pre‐ and post‐activity), we found that well‐being was significantly higher for both sets of students after the activity. Overall, this approach offers a model for combining active learning with museum collections to provide experiential research opportunities that highlight the power of scientific collaboration.

## Graphical abstract

We describe an authentic research activity (ARE) activity that leverages digitized diet data from natural history collections to provide students with collaborative research experience across any learning environment. Using magnified photographs of frog stomach contents collected in the Peruvian Amazon, we designed an open‐source “bowl game” competition that challenges students to identify, measure, and compare diet items across vouchered frog specimens (“Batrachian Barf Bowl”). This activity offers a model for combining game‐based learning with museum collections to provide experiential research opportunities highlighting the power of scientific collaboration.

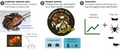

## 1 INTRODUCTION

Providing opportunities for student participation in ongoing scientific research is a foundational part of science education. Students gain valuable, practical experience in their chosen career fields prior to entering the job market post‐graduation (Cooper et al., 2019; Hernandez et al., 2018, and references therein) and often cite these experiences as critical to their retention in science, technology, engineering, and math (STEM) fields (Nerio et al., 2019 and references therein). For many students, their first exposure to scientific research occurs in undergraduate courses, where they perform laboratory experiments, partake in fieldwork, and generate their own scientific hypotheses. Multiple models have emerged for providing research experiences grounded in active data collection (authentic research experiences, or AREs), demonstrating important positive impacts for broadening participation and promoting retention in science (President's Council of Advisors on Science and Technology, 2012; Spell et al., 2014). However, there are numerous challenges associated with creating such activities and providing these types of research opportunities.

Limited access to research groups is perhaps the largest barrier to active research experiences for undergraduate students. Traditionally, research outside the classroom setting favors students who do not have additional work or familial obligations and those who come from financially privileged backgrounds (Sidlauskas et al., 2021). Additionally, these research experiences are often limited in the amount of personnel they can accommodate or are not tailored for larger groups (Hernandez et al., 2018). Creating research‐based activities like course‐based undergraduate research experiences (CUREs) can be an extremely time‐consuming undertaking for the instructor, even with the resources that help reduce the barriers to implementation (Govindan et al., 2020). Therefore, when CUREs are not practical, it can be beneficial to both students and instructors to incorporate the use of real data from on‐going research projects and natural history collections, especially when results can later be used for future research analyses.

Natural history collections can be a powerful tool for creating meaningful experiences that connect students with otherwise abstract ideas of the natural world (Monfils et al., 2017; Sidlauskas et al., 2021). For example, field guides are a widely popular item within and outside biology circles, yet many students are unaware of how the data necessary for each species description is actually collected and stored. Natural history collections provide students the ability to explore huge numbers of records and data associated with individual species, and therefore the opportunity to make conclusions about what a certain animal may prefer to eat, where it lives, how big it gets, and so on. However, natural history‐based research and classes pose a particularly unique set of virtual challenges in that these types of experiences often rely heavily on hands‐on activities, physical observation of specimens, and field trips. The recent surge in the digitization of collections has made these resources substantially more accessible, facilitating their widespread use in biodiversity‐based courses, lectures, and research experiences (Miller et al., 2020; Monfils et al., 2017; Walsh et al., 2019). But, the ability to observe and discuss observations with fellow classmates and instructors (as one might do in a classroom with lab stations) is more limited on electronic communication platforms. In turn, adjusting to these “new” social interaction norms during the pandemic led to decreased student collaboration and added the need to foster a sense of community among both students and faculty alike (Walsh et al., 2021).

It is well known that virtual learning can suffer from a lack of student engagement, reduced access to hands‐on resources, and a decreased sense of community and camaraderie (Faulconer & Gruss, 2018; Hsu & Rowland‐Goldsmith, 2021; Kebritchi et al., 2017; Wolinsky, 2021). Field biology instructors were especially worried about reduced inclusive practices and learning outcomes during the first COVID‐interrupted semester (Barton, 2020). Student interactions beyond the scope of virtual labs and lectures were limited and further reduced by stay‐at‐home orders, leaving many students feeling even more isolated during online courses (Elmer et al., 2020; Mack et al., 2021). Despite these challenges, the pandemic encouraged educators to explore activities that make science more accessible and engaging (Wolinsky, 2021).

**FIGURE 1:**
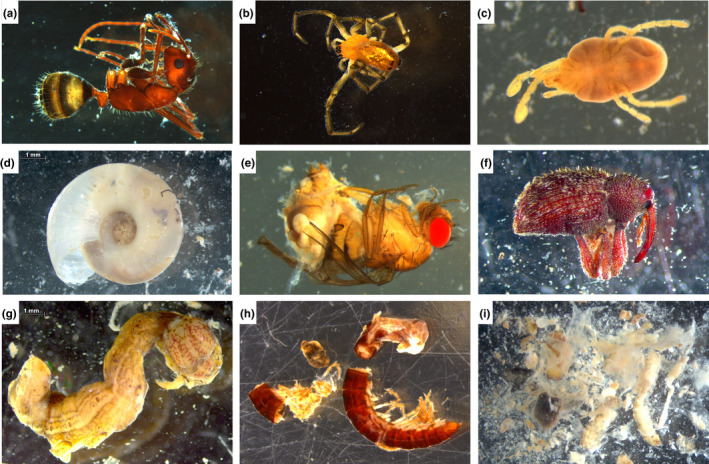
Photographs demonstrating the range of prey items in frog stomach contents that students encountered in the Batrachian Barf Bowl activity. Taxonomic identifications: (a) Hymenoptera (ant), (b) Araneae (spider), (c) Acari (mite), (d) Gastropoda (snail), (e) Diptera (fly), (f) Coleoptera (weevil), (g) Lepidoptera (caterpillar), (h) Polydesmida (millipede), and (i) unidentifiable invertebrate parts

Here, we provide an open‐source set of digitized natural history data associated with vouchered museum specimens and a new activity that addresses each of these challenges for an upper‐level biodiversity course. To unite ARE goals with natural history collections, this activity leverages data from an active research project on the evolution of diet niche space in frogs collected in the Peruvian Amazon and vouchered at the University of Michigan Museum of Zoology. In this research project, the stomach contents from thousands of frogs were collected through gastric lavage and photographed under dissecting scopes prior to DNA metabarcoding and sequencing (Figure 1; data available on Deep Blue Data [https://doi.org/10.7302/30be‐pe71]). Because frogs do not chew their invertebrate food, these photographs contain many well‐preserved prey items that can be identified to taxonomic order (Figure 1a‐h). Other prey items are more fragmentary if they had been in the stomach long enough to be partially digested (Figure 1i), representing an authentic continuum of data quality that biodiversity researchers face when studying the ecology and evolution of predator–prey interactions.

**FIGURE 2:**
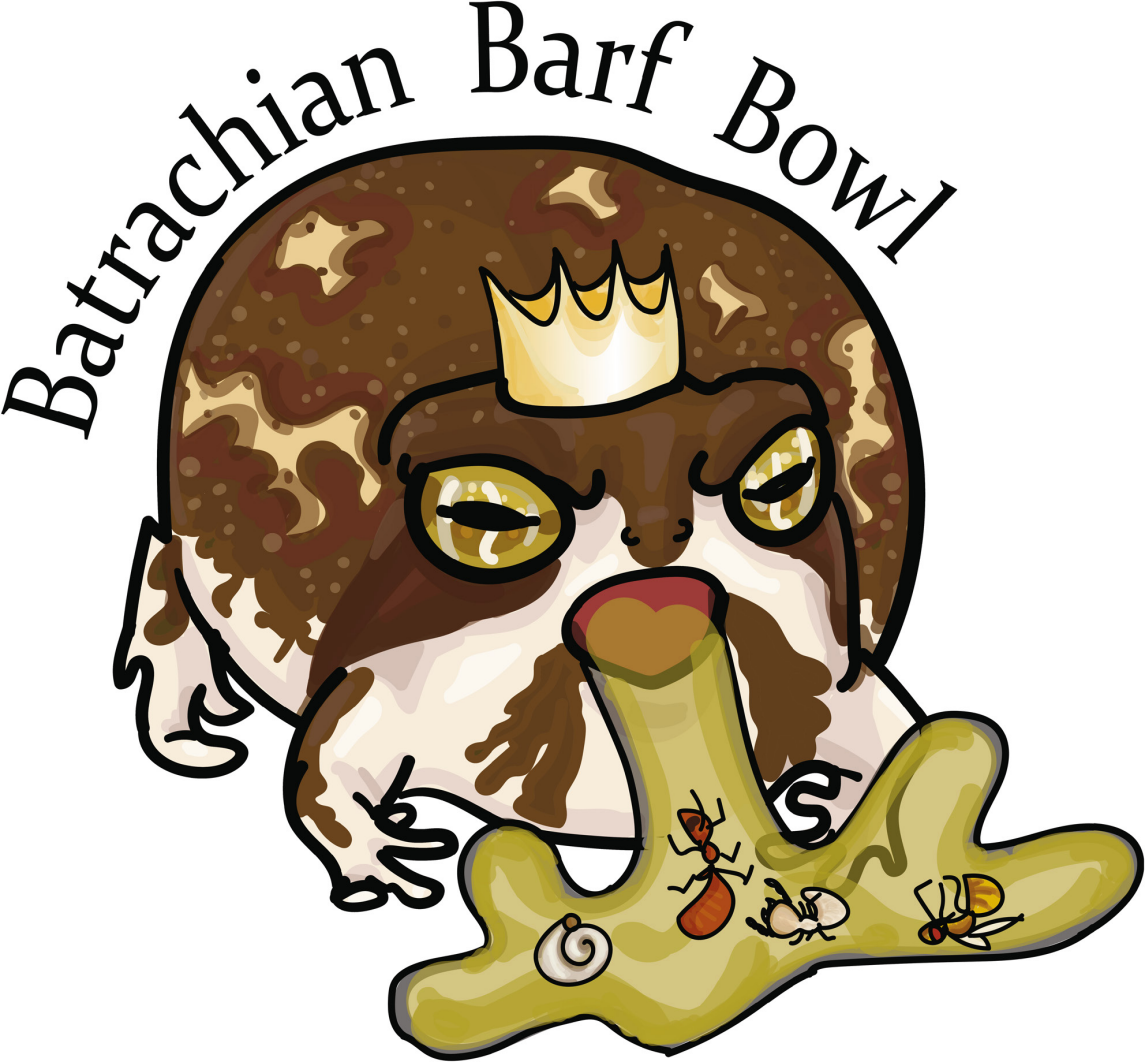
Graphic created as the “official” logo of the Batrachian Barf Bowl that can be used for future iterations of the activity. Designed by Natalie Claunch

Using these photographs, we present an open‐source “bowl game” friendly competition that challenges students to identify, measure, and compare diet items across vouchered frog specimens. Then, we present our findings of the efficacy of inter‐institutional activities and the tools for instructors to implement this inter‐institutional activity using the provided dataset of frog diet samples or to use this framework with an alternate dataset. To demonstrate learning outcomes, we ran this activity with 39 herpetology class students from the University of Michigan and the University of Notre Dame with pre‐ and post‐activity assessments of both accuracy and student experience. To set the tone for the activity, we chose a name that is engaging and informative: the Batrachian Barf Bowl. The term *Batrachia* refers to the amphibian clade that includes frogs and salamanders and therefore indicates our focal organisms. We also embraced the friendly rivalry between the two institutions by referring to the event as a “bowl.” Post‐season football games among invited, top‐ranked colleges in the United States/Canada are frequently referred to as bowl games, in reference to the original “bowl” shapes of the stadiums (https://bowlseason.com/news/2020/10/23/‐bowl‐season‐announced‐as‐new‐name‐of‐college‐footballs‐postseason.aspx). To capitalize on the appeal of alliteration, we called the frog diet samples “barf” since the stomach flushing process is akin to inducing frogs to vomit. To further foster a sense of fun, we commissioned a logo for the Batrachian Barf Bowl that can also be used for future iterations of the activity (Figure 2; courtesy of Natalie Claunch).

By making our classroom activity a friendly competition between two herpetology classes, we aimed to harness the competitive and fun spirit between rival universities to enliven a research experience. While many students initially enter STEM majors due to a childhood love for the subject matter, demanding undergraduate introductory STEM classes, imposter syndrome, and university elitism can discourage students from pursuing a career in subjects they once found interesting and engaging (Koenig et al., 2012). By placing an ARE in this framework, we remind students that research can be both fruitful and engaging, while still professionally moving them forward in their careers as new researchers.

## 2 METHODS

### 2.1 Activity goals

We designed this laboratory activity around six main goals with specific learning objectives:

1. Authentic research connection: To understand common challenges and opportunities facing scientists who study patterns and processes driving biodiversity on Earth, including problems outside of their exact area of expertise and to which there is no single right answer.
2. Appreciation for the value of biodiversity collections: To recognize the kinds of questions that can be answered with museum specimens, highlighting the excitement of “biodiversity discovery” as some of the Neotropical invertebrates may be undescribed species photographed for the first time.
3. Understanding the scale of “big data” projects: To learn data management and scientific workflow skills essential for large‐scale datasets.
4. Identification of unfamiliar organisms: To practice using available keys (dichotomous or otherwise) as identification resources for “unknown species.”
5. Collaboration for successful scientific inquiry: To provide an experience in scientific collaboration and networking to solve challenges too big for any one person to solve alone.
6. Foster a sense of connection among students: To improve student well‐being through inter‐institutional course collaborations.

Within the Ecological Society of America's (ESA's) Four‐Dimensional Ecology Education (4DEE) Framework, this activity covers the dimensions of Core Ecological Concepts and Ecology Practices (i.e., methodological skills of ecology) as it exposes students to using dichotomous keys to assess realized diet niches across species (Berkowitz et al., 2018).

### 2.2 Participants

Courses in herpetology (the study of amphibians and reptiles) were offered concurrently at the University of Notre Dame (Notre Dame, IN) and the University of Michigan (Ann Arbor, MI) in the second semester of the 2020–2021 academic year. There were 12 students in the University of Notre Dame Herpetology class, including eleven seniors and one junior. Nine students had declared majors in a field of biology (Environmental Sciences or Biological Sciences), two were majoring in non‐biology STEM areas, and one was not majoring in a STEM area. There were 27 students in the University of Michigan class, including 23 seniors and four Master's students. All undergraduate students had declared majors in fields of biology (7 distinct majors), and all graduate students were pursuing degrees in the Environment and Sustainability program (Supplementary Material S1).

Notre Dame had fully in‐person classes for the 2020–2021 academic year, whereas courses at Michigan were fully remote and occurred on Zoom, a virtual meeting platform (Yuan, 2020). Because many Michigan students were not on campus during that year of remote instruction, some students were in time zones other than Michigan's Eastern Time Zone. To accommodate these students, the herpetology course at Michigan offered the option of asynchronous participation in lectures and lab by viewing video recordings at a time convenient to the students (see Supplementary Material S2 for asynchronous instructions). On the day of this activity, Michigan students were strongly encouraged to participate synchronously to experience the social aspect of the lab, but students who were unable to do so could still engage with the other components of the activity asynchronously. Since Notre Dame had fully in‐person instruction for the Spring 2021 semester, students in that course were asked to not come to the classroom that day, and instead attend virtually via Zoom.

### 2.3 Activity methods

**FIGURE 3:**
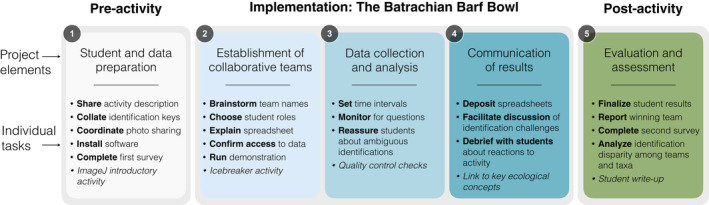
A conceptual framework for the activity workflow. By following the project elements in numerical order, individual tasks are easily reproduced by educators implementing the activity in their learning environments. Italicized tasks are recommendations for future implementations

We began the activity with a brief introduction to the class instructors and a research project from which the data was collected (Supplementary Material S2; all project stages are shown in Figure 3). Students were then manually assigned to breakout rooms on Zoom (Yuan, 2020), such that each group contained students from both universities. Students were given 5 min to introduce themselves to their group members and decide on a team name; we encouraged students to select a herpetology‐themed name. Then, we reconvened together to have groups share their selected team names and discuss any questions that had arisen in the breakout rooms. We then directed students back to their group breakout rooms to begin the activity.

Every group was assigned a folder on a shared drive within Google Drive (Google, 2021), although other cloud‐sharing platforms that can host large amounts of data would work equally well. Within each group folder were 24 subfolders, each of which contained the photographs of stomach contents from an individual frog (250–300 available photos, corresponding to 1–2GB, per group). Group folders contained diet photos from multiple frog species that vary in ecological similarity. These group folders were not identical, containing some frog individuals present in other group folders to assess the repeatability of identification and others that were unique to a particular group. We ensured that each group folder contained samples that ranged in the degree of digestion from pristine prey (Figure 1a–g) to fragmentary items (Figure 1h,i). Also included in the team folders was a template spreadsheet for students to input data that they collected from photos (Supplementary Material S3). We chose to use a Google sheet for data entry, as this allowed all group members to access and edit the document synchronously.

Students were instructed to download and install the free software, ImageJ (Schneider et al., 2012) on their computer prior to the start of class. We included instructions on how to use the software in the lab handout (Supplementary Material S1). Using the ImageJ program, students opened photographs for analysis. For each photo, we asked them to identify every prey item to a higher level of taxonomic classification (e.g., class and order), count how many of each prey type were present, and take basic linear measurements of length and width. Researchers take these two measurements to later calculate the volume of each prey item. To facilitate the identification of prey items, we provided students with multiple published identification keys to organisms that frogs are expected to eat, such as arthropods. Each photograph included a scale bar, which students used to calibrate the measurement tool in ImageJ. Students were given 45 minutes to identify prey items and record data. During this time, a graduate student instructor periodically stopped by the breakout rooms to check on groups and answer any questions that may have arisen as the activity was underway.

Ten minutes prior to the end of the class period, we brought all of the students back together in the main virtual classroom and asked them to share their impressions, the challenges of the activity, and the unexpected discoveries within the photos. Some students stayed after the end of the class period to continue the discussion.

### 2.4 Prey identification accuracy

Every group submitted the spreadsheet in which they entered data (Supplementary Material S4). After the activity, we checked the identifications of prey items made by students against a master identification spreadsheet generated by the original project researchers. To determine the winners of the Batrachian Barf Bowl, we scored groups based on the number of identifications that they made that were reasonably accurate and included count and measurement data. For identifications, we required scientific names to be accurate to the order level (e.g., Orthoptera and Coleoptera) within Arthropoda and class‐level in other clades. We accepted common names (e.g., wasp, ant, and spider), in addition to scientific names, but did not mark overly broad answers (e.g., larvae) as correct. To examine the power of this activity for generating usable data in an application of community science, we tallied how many identifications were correct and how many were misidentified, as well as the number of false negatives and false positives, across invertebrate clades represented in the photographs. In this context, a false negative occurred when students failed to observe a particular prey item in a sample. False positives occurred when students misidentified disarticulated components of one prey item as two separate items.

### 2.5 Assessment methods

To assess the learning outcomes, we used validated instruments to survey students the day before the activity and 2 weeks after the activity (Supplementary Material S5). To maintain anonymity from their instructors, the author who was not affiliated with either class (LLW) received ethical approval from the Institutional Review Board [DDPSC IRB 2021_04 exempt based on HHS regulations 45 CFR 46.104(d)(1) and 45 CFR 46.104(d)(2)] to distribute surveys. Before and after the activity, we used a 4‐point Likert scale to measure student self‐efficacy (Schwarzer & Jerusalem, 1995), science identity (Chemers et al., 2011; Estrada et al., 2011), and scientific community objectives value (Estrada et al., 2011) and we used a 5‐point Likert scale to measure student mental well‐being (Tennant et al., 2007). After the activity, we also surveyed students using the LCAS collaboration scale (Corwin et al., 2015). We used a 4‐point Likert scale for all the instruments except well‐being because a fifth “neutral” option is not recommended for questions in which students are likely to have an opinion, even if slight (Bandalos, 2018).

To collect feedback on the activity from students, our survey also included four open‐ended questions (Table 1). Responses for each question were thematically analyzed using inductive analysis (Braun & Clarke, 2006) and coded independently by two authors, with initial inter‐rater reliability of 0.796 Cohen's Kappa. Coding was reconciled through open discussion between the two coders.

**TABLE 1:** Four open‐ended questions were distributed to students after the Batrachian Barf Bowl activity

**Assessment questions**
Did you enjoy meeting herpetology students from another university? Explain why or why not. How much time would you recommend to be spent on cross‐university activities like this? What did you like most about Barf Bowl? What did you like least about Barf Bowl?

### 2.6 Assessment analysis methods

Students who did not participate in both surveys (pre‐bowl and post‐bowl) were removed from the dataset so that a total of 7 Notre Dame students and 12 Michigan students were included in the analyzed dataset (response rate of 58.33% and 50%, respectively). Likert scale responses were converted to numbers (e.g., strongly disagree = 1, strongly agree = 4 for 4‐point scales; none of the time = 1, all the time = 5 for 5‐point well‐being scale), and student constructs (science identity, scientific community objectives, self‐efficacy, well‐being, and LCAS collaboration) were calculated by averaging the responses from the respective instruments. For example, a student's responses to the five science identity questions were averaged to calculate their overall science identity score.

We used the Mann–Whitney *U* test in SPSS 27.0 (IBM 2021) to compare the construct scores between institutions to identify variation (e.g., Notre Dame pre‐bowl science identity vs. Michigan pre‐bowl science identity). We also evaluated the student scores collectively for change between pre‐ and post‐activity measurements using paired *t*‐tests in SPSS.

## 3 RESULTS

### 3.1 Participation

All 12 of the Notre Dame students participated in the activity synchronously with 15 of the Michigan students. These 27 students were divided into eight groups that had three or four students. The other 10 Michigan students completed the activity asynchronously individually or in self‐selected pairs. The synchronous teams were instructed to select a team name and several of the asynchronous students also chose a moniker. This aspect of the activity allowed the students to flex their creativity and humor, with team names including Herpetologeniuses, TOADally Ribbitting, Barfonidae, Barf Brigade, Barftrachians, Bug Crew, and Frognatic.

### 3.2 Prey identification results

Together, students assigned an identification to 180 prey items. There was notable variation in the number of identifications that each synchronous group completed during the class period, with a mean of 11 identified prey items per group (range: *N* = 9–21). Asynchronous groups who were instructed to analyze a specific number of frog individuals and were not time‐limited made a mean of 17 identifications (range: *N* = 12–37). Of the identifications, the mean number that was correct was the same for both the synchronous and asynchronous groups (70%), although the standard deviation was higher for the synchronous group (±23%) compared to the asynchronous group (±10%).

Misidentified prey items were more common in the synchronous groups (mean: 32% of prey misidentified) than in asynchronous (mean: 21% of prey misidentified). False negatives (undetected in the photograph, *N* = 50) showed the opposite pattern, being less common in the synchronous groups (mean: 11% of prey items not documented) than the asynchronous ones (mean: 22% of prey items not documented). For some groups, false negatives might not be cases of unobserved prey, but rather unidentifiable prey. While we encouraged students to attempt an identification for all items, we were not explicit in stating that they should write “unknown” rather than omitting prey items for which they could not hazard a guess.

**FIGURE 4:**
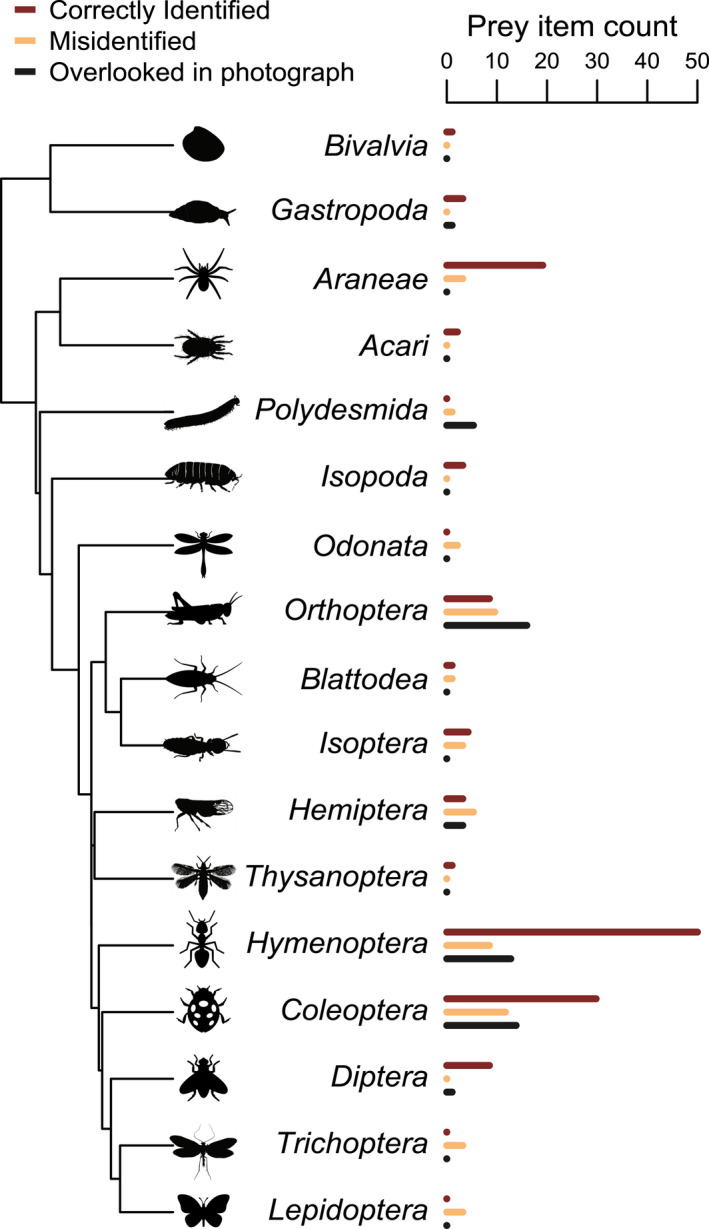
Student prey identification accuracy varied across taxonomic groups encountered in the prey item photos. Generally, spiders, hymenopterans (ants, wasps, and bees), coleopterans (beetles), and dipterans (flies) were identified correctly at a higher rate than other groups (red bars compared to orange bars). A non‐trivial number of prey items were overlooked completely in the photographs (black bars). Organism icons from phylopic.org

Some types of prey seemed to be more challenging for students, and accuracy varied by taxonomic order (Figure 4). For example, several photos included millipede (Diplopoda: Polydesmida) prey, however, these were never correctly identified and were most often false negatives (*N* = 4). One was misidentified as a Hymenopteran. We were surprised that caterpillars (larvae of Lepidoptera) were misidentified three times: once as a Diptera and twice as Tubellaria. After seeing this pattern in the results, we reviewed the identification keys that we had provided to students and realized that none of them included a caterpillar. Instead, the incorrect identifications that they selected were the most probable if a student strictly followed the key and did not have strong prior knowledge about larval lepidopterans. More generally, misidentifications tended to skew towards prey items from common orders being assigned to various uncommon orders that were provided on generalized keys, but not present in the dataset (a “rare bias” phenomenon also recognized in bird identification, Bouillard et al., 2019).

Snails with shells were easily identifiable, although the level of specificity varied among groups. A slug (Mollusca: Gastropoda: Heterobranchia) was not recorded and therefore was a false negative. Other taxa that were frequently identified correctly are spiders (Araneae, *N* = 18), beetles (Coleoptera, *N* = 28), and ants (Hymenoptera, *N* = 46).

### 3.3 Student assessment results

**FIGURE 5:**
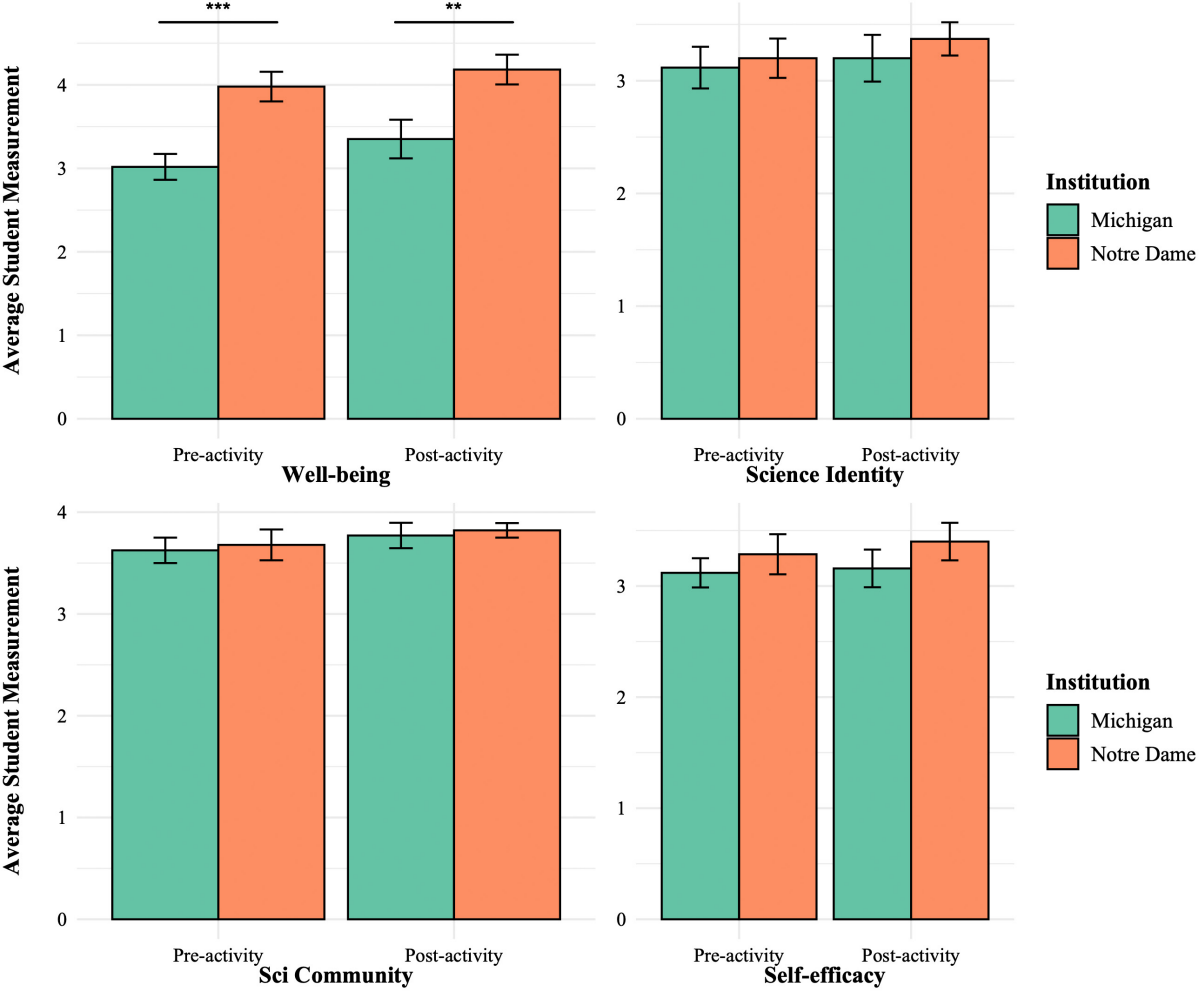
Students at both institutions self‐reported higher well‐being scores after the activity than they did before participating, although students at the University of Michigan (which ran obligately remote courses the entire semester; shown in orange) reported lower values overall in comparison to the University of Notre Dame (which ran in‐person the entire semester, with remote participation only for this activity)

We found no difference in science identity, scientific community, or self‐efficacy between the two institutions at either time point (all *U <* 41.5, *P* > 0.25, Mann–Whitney tests). However, we found well‐being was higher for Notre Dame students than for Michigan students at both time points (*U* = 6.0, pre‐activity *P* = 0.002; *U* = 11.5, post‐activity *P* = 0.010; Mann–Whitney tests; Figure 5; Supplementary Material S6). While all four student constructs increased in the post‐activity survey based on their collective averages, well‐being was the only construct that significantly increased in the post‐survey for student participants (*t* = −2.571, *P* = 0.019, paired *t*‐test). When asked if they enjoyed meeting herpetology students from another university, 88% of students indicated that they did, with 65% of students explaining why and 18% including limitations or constructive feedback (Table 2).

**TABLE 2:** Student responses to open‐ended questions in the post‐activity assessment, categorized by the major content of the response

Question	Theme	Respondents
*Meeting others*	Enjoyed BECAUSE	11
	Enjoyed BUT	3
	Did not enjoy	2
	Enjoyed	1
*Cross‐university activities*	More time	10
	More activities	5
	Alternative structure	2
*Best aspect*	Collaboration	10
	Research	10
	Novelty	5
*Greatest challenge*	Short time allotment	8
	Intent of activity	6
	Remote interaction	6
	Identification ambiguity	2

Students who enjoyed meeting other herpetology students included explanations that cited collaboration, shared interests, and hearing new perspectives. For example, one student from Michigan wrote,

I enjoyed it because it was interesting to see how their studies compared to ours and I know in my group personally, one of the members from the other university was even able to offer tips that I hadn't encountered from others in my class yet.

Students who enjoyed meeting other students but provided constructive feedback indicated that they would have liked more time for the activity or they felt the activity was limited over Zoom. One student from Notre Dame wrote,

Yes, I thought it was interesting to learn with students from another university, but I don't think I'd want to do the same activity over zoom again. I'd prefer for the class to be in person or perhaps a different style of competition.

The two students who did not enjoy meeting other herpetology students had similar feedback (*limited time, Zoom*), with one writing,

Not particularly. It was pretty awkward to meet people on Zoom, so we didn't have the ability to form a good relationship before working together.

When asked how much time they would recommend be spent on activities like this, 59% of students indicated more time, with a median time of approximately 2 hours (Table 2). Rather than provide a time length, some students indicated that they would recommend such activities implemented multiple times during the semester. One Michigan student wrote,

More than we do! This is the first time I've ever done something like this and I think it was really cool!

Two students suggested alternative, in‐person activities. For example, one student wrote,

Once would be a fun exercise, or in a different time, meeting together again for field trips would be nice.

When asked what they liked most about Barf Bowl, collaboration and research were each mentioned in 55% of responses, and the engagement or novelty of the activity was mentioned in 28% of responses (Table 2). One student wrote,

I really liked contributing to real research. I have not had a chance to do any of my own research as a full‐time student with an off‐campus job, so this was an opportunity that I was very excited for. (*Research*)

Another student enjoyed “doing a fun new activity with other students in a competitive environment” (*Collaboration; New Activity*).

When asked what they liked least about Barf Bowl, 47% of students felt the activity was too short. One student provided constructive feedback, writing,

I think that the Barf Bowl could be improved by having a short homework activity using the ImageJ software a few days before the lab. I think a guided tutorial that will help us know if we are using the software correctly (like calibrating the measurement units) would make the experience go more smoothly.

Approximately 35% of students indicated that they disliked that the activity was remote, and 35% of students were unclear about the intent of the activity (Table 2). For example, one student wrote,

It felt less like a competition and more like we were just helping with research.

Two students indicated that they did not enjoy the challenge of identifying organisms with which they were not familiar.

## 4 DISCUSSION

In this paper, we present a unique inter‐institutional undergraduate educational activity that engages students with real data and provides the opportunity to work collaboratively with peers. The majority of students enjoyed meeting herpetology students from another institution, indicating the activity was successful in forming connections across institutions. The similar measurements of science identity, the scientific community, and self‐efficacy at both institutions suggest the two classes had similar student audiences who would pair well together. Additionally, we found that, collectively, the well‐being of student participants increased after the Barf Bowl, an affective goal for our collaborative activity. However, since we did not survey a control group, such as a herpetology class that did not participate in Barf Bowl, we cannot conclusively attribute this increase to participation in the activity. We also observed significantly higher well‐being in the class that was conducted in‐person, which may be a side effect of campuses that held classes in‐person. Because of our small sample size (which is common in small enrollment natural history courses), we recommend interviews as a follow‐up to better understand the impact that in‐person classes might have on the well‐being of students in natural history and biodiversity courses.

While we present this activity within an inter‐institutional framework, it can also easily be implemented with just one class, either in person or virtually, to achieve the first five of our learning goals. In providing the prey photographs and instructional materials, we intend to offer a flexible activity concept and we encourage instructors to modify the implementation of the activity to best suit the needs of their class and situation.

### 4.1 Benefits of virtual research

Providing undergraduate students with the opportunity to conduct genuine research is a powerful tool for the retention of students in STEM fields (Nerio et al., 2019 and references therein). Research experiences can provide a number of benefits beyond increasing scientific literacy, such as increased confidence and a sense of belonging and community. Unfortunately, access to these types of opportunities, either through CUREs or working in a research lab, are not equally available to all students. Availability of course‐based research experiences may be limited by the financial position of the higher education institution, as many biology‐related CUREs require consumable materials or reduced class sizes. The socioeconomic status of students can also affect their ability to participate in research outside of the classroom, as students from less privileged financial situations may be obligated to spend their time working a paid job rather than volunteering in a laboratory (Sidlauskas et al., 2021). For similar reasons, many students in biology and environmental majors do not have the opportunity to conduct field work abroad or even locally. However, research experiences that use real data collected from scientific expeditions allow students to work with data and organisms that may not have been otherwise accessible. Participating in classroom activities such as the Batrachian Barf Bowl can “transport” students to far‐away and remote field sites, giving them the chance to experience what data collection and research skills would be needed to conduct a field‐based research project. The Batrachian Barf Bowl provides this experiential opportunity and can easily be implemented independently of institutional funding since all of the required materials are open‐source.

### 4.2 An emphasis on collaboration

In their responses, students highlighted the benefits of meeting other students who shared similar interests but also offered alternative perspectives on herpetology. These observations could be expanded in future Barf Bowls by instructors to highlight the importance of diversity in scientific collaborations. It is unusual for authentic research activities to highlight the importance of collaboration in research (Spell et al., 2014). In an age of big data, it is increasingly the norm for research teams to have several to dozens of members to deal with the scale of data and have the diverse experiences required to integrate different data types. The research project that inspired Barf Bowl is an excellent example of the teamwork needed to gather and analyze large datasets, using methods ranging from morphological identification to molecular metabarcoding and requiring expert knowledge of amphibians and arthropods. Expanding on this topic would also be an opportunity for instructors to provide students with personal examples of successful collaborations they have with scientists at other institutions, thereby tangibly highlighting the real‐world applications of developing collaborative skills.

### 4.3 Extensions into ecology and evolutionary lessons

The activity framework that we present in this paper is highly flexible, as evidenced by the multiple possible extensions that we outline. It would be easy to apply the concept of an inter‐institutional ARE to other datasets that might be more relevant to the course content. In our dataset, each stomach contents sample can be linked back to an individual frog (Supplementary Material S7), allowing students to investigate a wide range of questions about frog dietary ecology. For example, students can examine whether different frog species have similar diets or whether they eat prey of different sizes. They could also be asked to examine whether frog diets appear to be more influenced by evolutionary history (as defined by their taxonomic classification; Supplementary Material S7) or by the microhabitat associations of the species (e.g., arboreal, aquatic, and terrestrial). These and other questions could be addressed by having students calculate some simple metrics of diet, like Simpson's Diversity Index as a measure of dietary breadth, for frog individuals or species. Metrics could be weighted by either the number of each prey item eaten or the volume of each prey type, estimated as the volume of a prolate spheroid using length and width (Vitt & Caldwell, 1994).

A further extension could ask students to reflect on the potential insights to be gained from integrating other types of information about each frog individual or species, such as head morphology, intestinal microbiome, or chytrid fungal infection status. Reflecting on research questions such as these can help students appreciate that these types of projects could be undertaken because the diet samples come from “super specimens” with auxiliary ecological data (Webster, 2017) that are deposited in natural history collections. These vouchered specimens were collected and preserved in such a way as to make them maximally useful to current and future researchers, and this approach could be applied to any collection of heterotrophs with identifiable prey items.

### 4.4 Virtual vs. in‐person learning

As most educational institutions return to in‐person learning, instructors intending to use this inter‐institutional activity framework will need to consider how to connect the two classes and the potential logistical implications for students. In our implementation of this activity, Notre Dame herpetology students were asked to join via Zoom, in contrast to how they normally attended courses during the semester. This modality meant that they needed to be in a location suitable for a video meeting that required their participation. For many of the students, this location was their off‐campus housing because there were limited locations on campus that fit the requirements. It is possible that changing to a virtual setting imposed hardships on students who had to be present on campus for other courses before or after this activity. Future implementations of this activity should be mindful of possible challenges for students to remotely join a class when this is not the normal procedure for them. Instructors should consider locating and reserving spaces on campus suitable for video calls for groups of students.

If the two participating institutions are geographically close to each other, there is also the possibility of having this be an in‐person activity, if time and transportation permit. This would address one of the areas of feedback we received from students, which was that they wanted more interaction with students from the other class. Several students also voiced the desire to have a joint field trip to look for wild amphibians and reptiles. These in‐person extensions are not necessary for a successful and impactful version of this activity but could be considered to further bolster inter‐institutional student connections.

### 4.5 Future recommendations

Overall, students would have benefited from a clearer introduction to the Barf Bowl. This introduction would provide a background on the research and its purpose, why they were analyzing research photos as a lab exercise, and why they were challenged to identify invertebrates in a herpetology course. The introduction should draw a clear connection for students between how their herpetology identification skills can translate to identifying other taxa.

It is possible that this activity currently has its greatest utility as an educational rather than research exercise. In order for it to generate usable data and be an effective community science project, additional preparation of the students to identify invertebrates would be required before photo analysis. This training could be in the form of scaffolding homework assignments that guide students through the use of arthropod identification keys prior to the formal exercise, thereby increasing their confidence and efficiency during class time. Such activities would also allow students to practice using the ImageJ software in advance of the teamwork. Another avenue for increasing the accuracy of prey identification would be to provide students with tailored arthropod identification keys. For this inaugural Barf Bowl, we used published generalized invertebrate identification keys. Key ambiguity resulted in the inclusion of clades that were not represented in any frog diets and the exclusion of others, such as millipedes. While we told students that these were not comprehensive keys for this project, it is likely that many of the incorrect identifications and false negatives resulted from a tendency of students to think strictly in terms of the identification keys. Two students responded in the post‐activity survey that they did not enjoy the activity because it was challenging to identify the prey items due to their unfamiliarity with invertebrates. A dichotomous key designed specifically for this activity might have alleviated some of this frustration. However, the imperfect keys supplied to students replicated the reality faced by most researchers when they embark on a new research project without complete knowledge or perfect methods, and underscores the fact that a major component of science is the process of discovery. Historical studies of frog diets relied on visual examination of stomach contents like these, and for the vast majority of species, these are the only data that exist on ecological relationships between predator and prey. The challenges of generating the types of diet data necessary for field guides, textbooks, and predator–prey research are often unappreciated by people who do not directly engage with the underlying research or data collection. Future iterations of this activity should explicitly contextualize this aspect of research and encourage students to embrace the problem‐solver mindset that is crucial to scientific discovery.

Based on student feedback, future iterations of this activity should be longer to allow students to process more samples. Prior homework assignments that prepare students to use ImageJ would also result in more time for sample analysis during the activity since students would not spend time learning a new software program. Additional time or a second session could be added to the activity for a more focused analysis of the results or to have students build an identification key for the prey items that the class observed.

## AUTHOR CONTRIBUTIONS

**Joanna G Larson:** Conceptualization (equal); data curation (equal); formal analysis (equal); funding acquisition (equal); investigation (equal); methodology (equal); project administration (equal); resources (lead); supervision (equal); writing – original draft (equal); writing – review and editing (equal). **Hayley Layne Crowell:** Conceptualization (equal); data curation (equal); investigation (equal); methodology (equal); project administration (equal); supervision (supporting); writing – original draft (equal); writing – review and editing (equal). **Lisa L Walsh:** Data curation (equal); formal analysis (equal); investigation (equal); methodology (equal); visualization (supporting); writing – original draft (supporting). **Alison Davis Rabosky:** Conceptualization (equal); formal analysis (equal); funding acquisition (equal); investigation (equal); methodology (equal); project administration (equal); supervision (lead); visualization (lead); writing – review and editing (equal).

## CONFLICT OF INTEREST

The authors of this manuscript declare no competing interests.

### OPEN RESEARCH BADGES

This article has earned an Open Data badge for making publicly available the digitally‐shareable data necessary to reproduce the reported results. The data is available at [[insert provided URL from Open Research Disclosure Form]].

## Supporting information


**Appendix S1** Participant demographics

**Appendix S2** Activity handout

**Appendix S3** Template diet data spreadsheet

**Appendix S4** Student activity results

**Appendix S5** Pre‐ and post‐activity student surveys

**Appendix S6** Student survey results

**Appendix S7** Frog taxonomic identification

## Data Availability

Photos of frog diet items used for this activity are available on Deep Blue Data (https://doi.org/10.7302/30be‐pe71).
