# Supplementary material for: The Batrachian Barf Bowl: An authentic research experience using ecological data from frog diets
Source: Ecol Evol. 2022 Jul 17;12(7):e9095. doi: 10.1002/ece3.9095 (PMC9288929; doi:10.1002/ece3.9095)
Supplement: Supplementary file 2 — Appendix S2 Activity handout [file ECE3-12-e9095-s006.pdf]

Name: University of Michigan/Winter 2021

LAB #6 - BATRACHIAN BARF BOWL

Lab Goals

Today, we are learning about an active research project at the forefront of ecology and evolution in herpetology. Modeled after the HHMI initiative "Authentic Research Connection" that some of you may have taken here at UM, you are going to analyze real data in an ongoing Herpetology Division research project so you can see some of the challenges and opportunities facing scientists who want to better understand patterns and processes driving herpetofaunal diversity. Which frog species are generalists, and which are specialists? When species specialize, are there some prey types that have higher representation than others? Can two species that specialize on the same prey type live sympatrically?

Format of Exercise

This lab will be both a cross-collegiate scientific collaboration as well as competition! Herpetology classes from both University of Michigan and Notre Dame will combine powers to tackle a huge but incredible data set of frog diet samples from the neotropics. Upon the start of the lab period, you will be divided into teams of 4 (2 UM students and 2 ND). Teams will then compete to identify as many organisms as possible in the provided frog diet photos within the allotted time period. This lab will be mainly conducted on a laptop computer using the program Image J (please download it from this website prior to lab). We will provide two resources: 1) a standardized Google spreadsheet in which your group will enter your data and 2) a pictorial guide to some of the major orders of invertebrates that you might encounter (online key here). Then, you will download photographs of frog stomach contents to roughly assess the biomass of each major prey type (instructions below) so that we can analyze - as a group - how frogs partition diet resources across space. Yes, this exercise will be challenging! The key is to keep a broad perspective and do the best you can. At the end of the lab period, your group will turn in a single Google spreadsheet (file named with your team members' names) with your data in it. A premade Google spreadsheet, Invertebrate Guide, and Diet sample photos can be found in this Google folder.

How to analyze photos

1. In ImageJ, go to [**File → Open**] and select your first photo for analysis.
2. Using the "straight line" tool in the toolbar (5th from the left), draw a straight line across the scale bar in the photo.
3. Once you've drawn this line, go to **[Analyze → Set Scale].** In the window that pops up, enter the length of the scale bar in the box for "Known distance" in terms of millimeters (mm). Most scalebars will be in units of mm already, but some might be in cm, so take note and convert as needed. Set the "Unit of length" to mm. Click ok.
4. Using the "straight line" tool, draw a line along the length of the prey item. Sometimes the prey item will be bent in such a way that a segmented line will more accurately measure the length. To switch between the straight and segmented line tool, right click on the icon

in the toolbar (5th icon from the left) and select which option you would like. Once you have drawn this length line, go to [**Analyze → Measure**] or use the keyboard shortcut to get a length. You can leave the "Results" window open as it won't write over your previous measurements.

5. Repeat this measurement process for the length of the prey item, measured at the widest point of the body.
6. Enter or copy and paste the length and width measurements into the Google sheet, along with the identification of the prey item. If there are multiple individuals of the same prey species and they are approximately the same size, you only need to measure one. Enter the number of each prey species in the "Count" column of the spreadsheet.

Asynchronous Students

In order to get full credit for this lab, you will need to measure your own frog diet sample independently and turn in your spreadsheet on Canvas. The number of samples you are required to complete will be determined based on the amounts each team measures during the synchronous lab period; this amount will be announced on Canvas to stay tuned.

References

We recognize that this is not an invertebrate zoology class and that most of these prey items may be new to you. That said, these are the real challenges that herpetologists face when trying to answer basic "amphibian biology" questions like these! There is no other way to get fine-scale information about what frogs eat than by analyzing information from their gut contents, either by coarse prey categorization or by DNA sequencing. When you read in a field guide that "Wood frogs eat insects, arachnids, worms, slugs, and snails," the only reason we know that is because someone did exactly what you are doing today. For some of the species you analyze in this lab, these may be the first known records of diet items, period!

Remember that invertebrates represent the vast majority of species diversity alive on this planet today - which makes them a critical food resource for amphibians. As in vertebrates, diversity is not distributed equally across taxonomy (see pie chart from lecture): most invertebrates are arthropods, most arthropods are insects, and most insects are beetles (Coleoptera), butterflies/moths (Lepidoptera), true flies (Diptera), and ants/wasps/bees (Hymenoptera). Don't forget that insects also have larvae, which usually look different from the adults!

Aquatic Macroinvertebrates

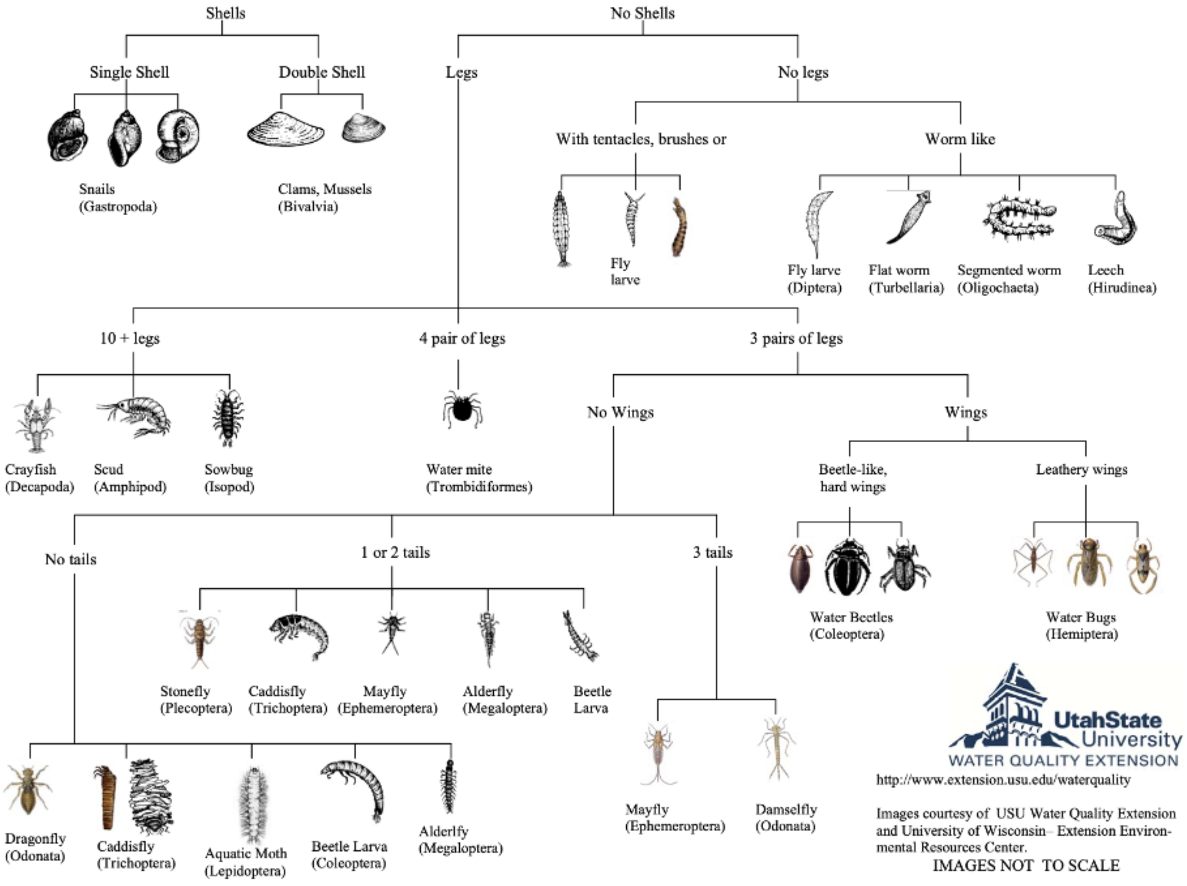

Terrestrial Macroinvertebrates

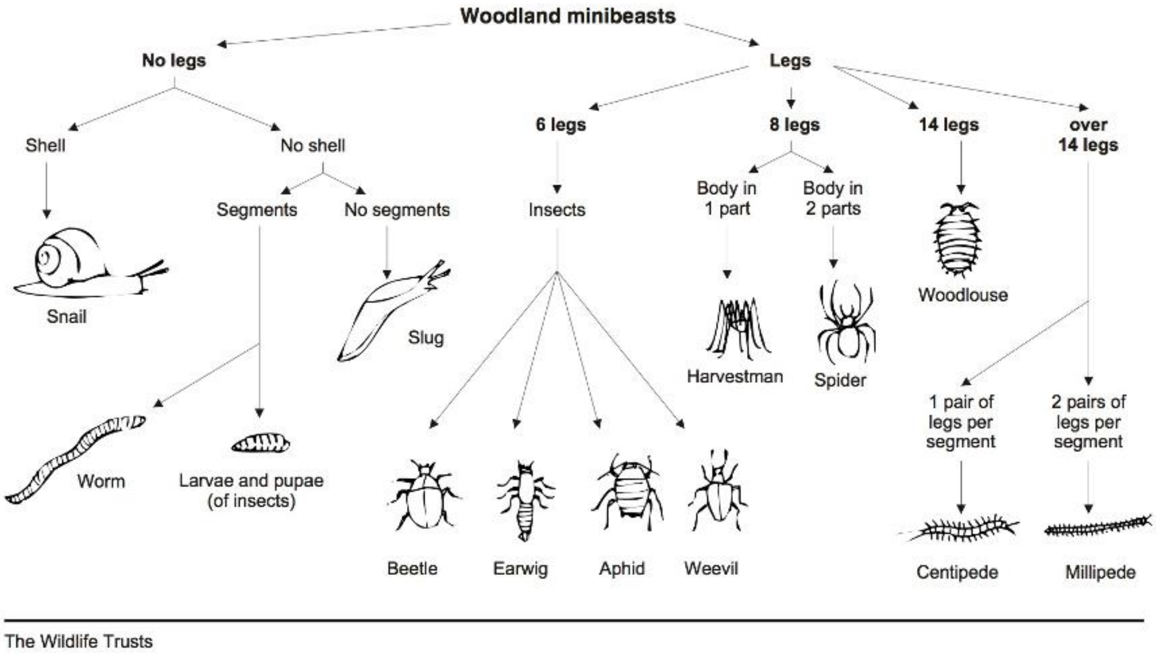

Color Drawings & Size Estimates

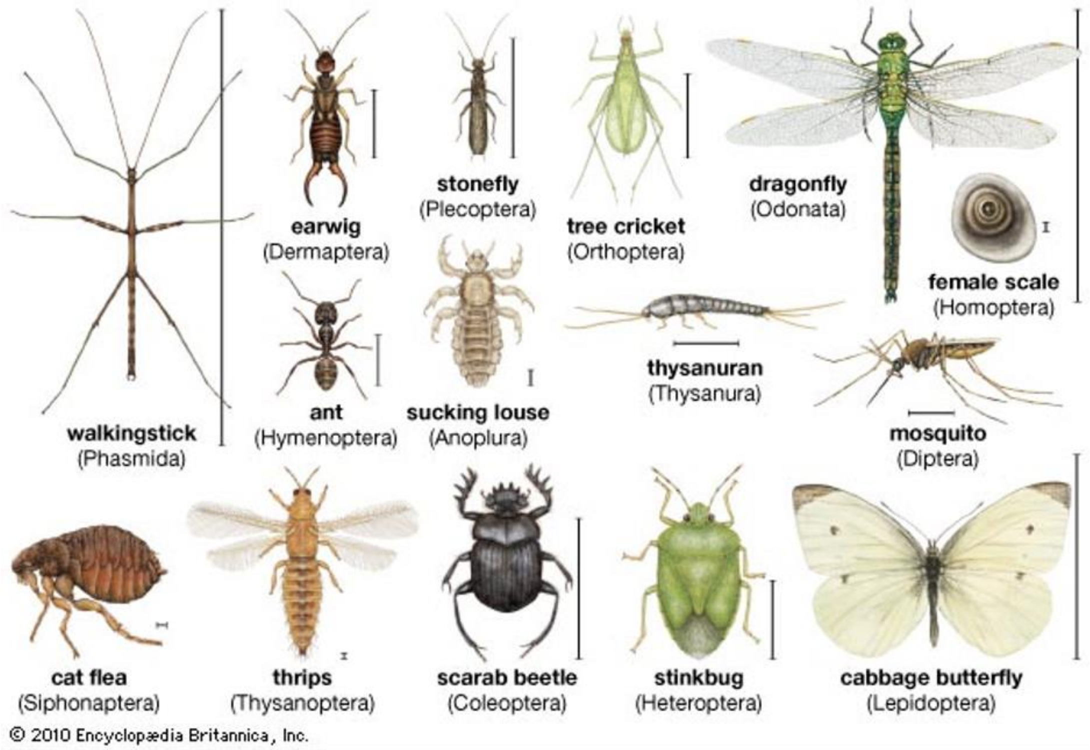

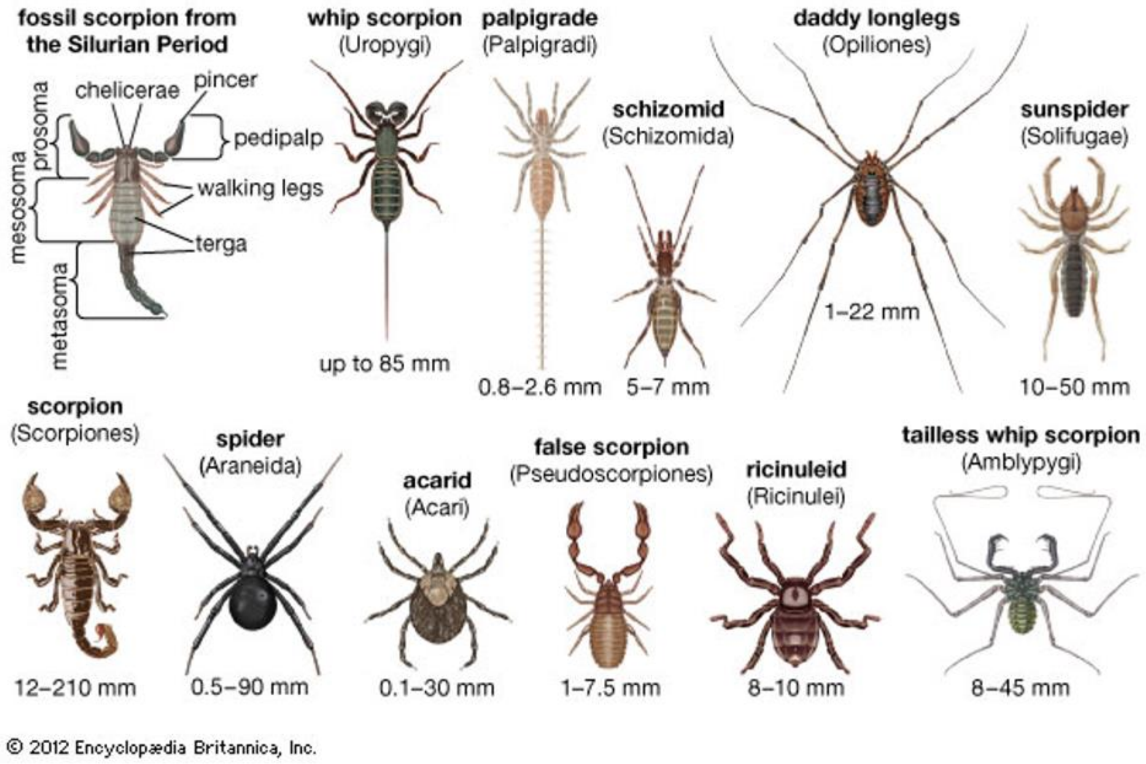

The Big Ten Insect Orders			
Order	Example	Common traits	Illustration
Hymenoptera ("Membrane Wings")	Ants, bees, wasps	Two pairs of clear, membranous wings, long legs, stingers	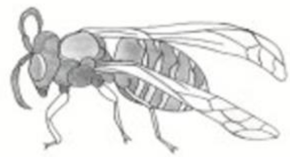
Diptera ("Two Wings")	Flies, mosquitos, gnats	One pair of regular wings and one pair of very small wings, sucking or sponge-like mouthparts	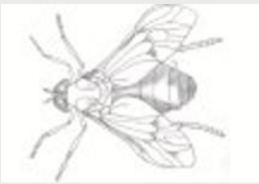
Lepidoptera ("Scaly Wings")	Moths, butterflies	Two pairs of scaly wings, large antennae, sucking mouthparts	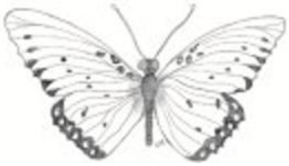
Coleoptera ("Sheath Wings")	Beetles, weevils	One pair hard wings, which coved top of body and meet in a straight line down the back, biting mouthparts	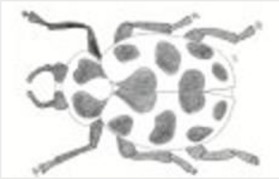
Orthoptera ("Straight Wings")	Crickets, grasshoppers, locusts	One pair of leathery wings in front (fold over the body when not in use), one pair of fan-like wings in back, chewing mouthparts	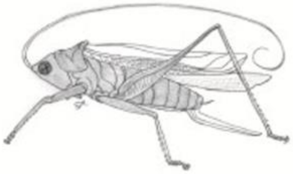
Homoptera* ("Same Wings")	Aphids, cicadas, treehoppers, leafhoppers	Two pairs of wings that are the same from base to tip, wings held like a tent over body when resting, often piercing mouthparts	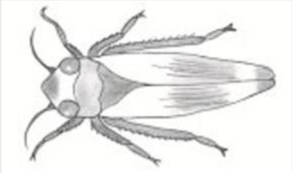
Heteroptera* ("Different Wings)<br><br>* Both are now "Hemiptera"	True bugs, water striders	Two pairs of wings: thick and leathery near body and thin at tip, wings fold on back forming a triangle behind the head, piercing snout	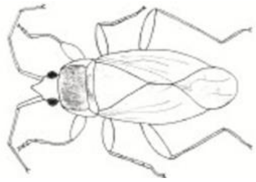

Blattodea (“Roaches”)	Roaches and termites	Two pairs of wings (leather or membranous), hard-bodied or soft-bodied, variable mouthparts	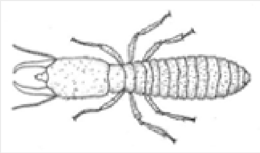
Odonata ("Born with teeth/Toothed Wings”)	Dragonflies, damselflies	Two pairs of wings, short antennae, biting mouthparts, **aquatic larvae**	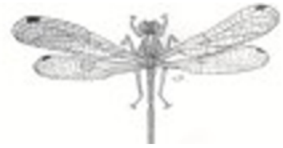
Trichoptera (“Hairy Wings”)	Caddisflies	Two pairs of hairy, membranous wings, long antennae, **aquatic larvae**	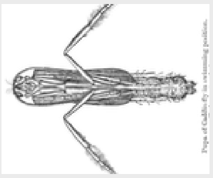
