# Supplementary material for: The Batrachian Barf Bowl: An authentic research experience using ecological data from frog diets
Source: Ecol Evol. 2022 Jul 17;12(7):e9095. doi: 10.1002/ece3.9095 (PMC9288929; doi:10.1002/ece3.9095)
Supplement: Supplementary file 5 — Appendix S5 Pre‐ and post‐activity student surveys [file ECE3-12-e9095-s003.pdf]

Herpetology - Presurvey

Start of Block: Permission

Q3 Please provide your First Name:

____

Q4 Please provide your Last Name:

____

Q2 Permission to Participate in a Research Study

Title: Herpetology across campuses

Contact Information: lwalsh@danforthcenter.org

Overview: This research is evaluating the effectiveness of a new herpetology activity. The researcher in charge of this study is Dr. Lisa Walsh, a postdoctoral researcher at the Donald Danforth Plant Science Center. She will make every effort to keep private all research records that identify you and will limit the identifiable information collected about you, but will request your name in order to compare survey responses over two time periods. Names will be converted to randomly generated identifiers before any information is shared with other members of the research team. She may publish the results of this study. However, your name and other identifying information will be kept private.

Your information will be combined with information from other students taking part in the study. When Dr. Walsh writes about the study to share it with other researchers, she will write about the combined information gathered.

By clicking the button below, you acknowledge that your participation in the study is voluntary,

you are 18 years of age, and that you are aware that you may choose to terminate your participation in the study at any time and for any reason.

- ☐ I consent to participate in this study (1)
- ☐ I do not consent to participate in this study (2)

Skip To: End of Survey If Permission to Participate in a Research Study Title: Herpetology across campuses Contact Informat... = I do not consent to participate in this study

End of Block: Permission

Start of Block: Science identity

Q5 To what extent do you agree or disagree with the following statements:

	Strongly disagree (1)	Somewhat disagree (2)	Somewhat agree (3)	Strongly agree (4)
I have a strong sense of belonging to the community of scientists (1)	o	o	o	o
I derive great personal satisfaction from working on a team that is doing important research (2)	o	o	o	o
I have come to think of myself as a 'scientist' (3)	o	o	o	o
I feel like I belong in the field of science (4)	o	o	o	o
The daily work of a scientist is appealing to me (5)	o	o	o	o

Q6 To what extent to do following statements sound like you?

	Not at all like me (1)	Somewhat not like me (2)	Somewhat like me (3)	Very much like me (4)
A person who thinks it is valuable to conduct research that builds the world's scientific knowledge (1)	○	○	○	○
A person who feels discovering something new in the sciences is thrilling (2)	○	○	○	○
A person who thinks discussing new theories and ideas between scientists is important (3)	○	○	○	○
A person who thinks that scientific research can solve many of today's world challenges (4)	○	○	○	○

End of Block: Science identity

Start of Block: GSE

Q1 From your experience this current academic semester, to what extent do you agree or disagree with the following statements:

	Strongly disagree (1)	Somewhat disagree (2)	Somewhat agree (3)	Strongly agree (4)
I can always manage to solve difficult problems if I try hard enough. (1)	○	○	○	○
If someone opposes me, I can find the means and ways to get what I want. (2)	○	○	○	○
It is easy for me to stick to my aims and accomplish my goals. (3)	○	○	○	○
I am confident that I could deal efficiently with unexpected events. (4)	○	○	○	○
Thanks to my resourcefulness, I know how to handle unforeseen situations. (5)	○	○	○	○
I can solve most problems if I invest the necessary effort. (6)	○	○	○	○
I can remain calm when facing difficulties because I can rely on my coping abilities. (7)	○	○	○	○

When I am confronted with a problem, I can usually find several solutions. (8)	○	○	○	○
If I am in trouble, I can usually think of a solution. (9)	○	○	○	○
I can usually handle whatever comes my way. (10)	○	○	○	○

End of Block: GSE

Start of Block: MW

Q7 Please select the option that best describes your experience of each over the last 2 weeks

	None of the time (1)	Rarely (2)	Some of the time (3)	Often (4)	All the time (5)
I've been feeling optimistic about the future (1)	○	○	○	○	○
I've been feeling useful (2)	○	○	○	○	○
I've been feeling relaxed (3)	○	○	○	○	○
I've been feeling interested in other people (4)	○	○	○	○	○
I've had energy to spare (5)	○	○	○	○	○
I've been dealing with problems well (6)	○	○	○	○	○
I've been thinking clearly (7)	○	○	○	○	○
I've been feeling good about myself (8)	○	○	○	○	○
I've been feeling close to other people (9)	○	○	○	○	○
I've been feeling confident (10)	○	○	○	○	○

I've been able to make up my own mind about things (11)	○	○	○	○	○
I've been feeling loved (12)	○	○	○	○	○
I've been interested in new things (13)	○	○	○	○	○
I've been feeling cheerful (14)	○	○	○	○	○

End of Block: MW

Herpetology - Postsurvey

Start of Block: Permission

Q3 Please provide your First Name:

____

Q4 Please provide your Last Name:

____

Q2 Permission to Participate in a Research Study

Title: Herpetology across campuses

Contact Information: lwalsh@danforthcenter.org

Overview: This research is evaluating the effectiveness of a new herpetology activity. The researcher in charge of this study is Dr. Lisa Walsh, a postdoctoral researcher at the Donald Danforth Plant Science Center. She will make every effort to keep private all research records that identify you and will limit the identifiable information collected about you, but will request your name in order to compare survey responses over two time periods. Names will be converted to randomly generated identifiers before any information is shared with other members of the research team. She may publish the results of this study. However, your name and other identifying information will be kept private.

Your information will be combined with information from other students taking part in the study.When Dr. Walsh writes about the study to share it with other researchers, she will write about the combined information gathered.
By clicking the button below, you acknowledge that your participation in the study is voluntary,

you are 18 years of age, and that you are aware that you may choose to terminate your participation in the study at any time and for any reason.

- [ ] I consent to participate in this study (1)
- [ ] I do not consent to participate in this study (2)

Skip To: End of Survey If Permission to Participate in a Research Study Title: Herpetology across campuses Contact Informat... = I do not consent to participate in this study

End of Block: Permission

Start of Block: LCAS

Q13(unique to virtual classroom students): Did you attend the Barf Bowl activity live (synchronously)?

- ○ Yes (1)
- ○ No (2)

Q8 To what extent do you agree or disagree with the following statements for the Barf Bowl activity:

	Strongly disagree (1)	Somewhat disagree (2)	Somewhat agree (3)	Strongly agree (4)
1 (1)	○	○	○	○
I was encouraged to reflect on what I was learning (4)	○	○	○	○
I was encouraged to contribute my ideas and suggestions during class discussions (5)	○	○	○	○
I was encouraged to help other students collect or analyze data (6)	○	○	○	○
I was encouraged to provide constructive criticism to classmates and challenge each other's interpretations (7)	○	○	○	○
I was encouraged to share the problems I encountered during my investigation and seek input on how to address them (8)	○	○	○	○

Q9 Did you enjoy virtually meeting herpetology students from another university? Explain why or why not:

____

Q10 How much time would you recommend be spent on cross-university activities like this?

____

Q11 What did you like most about Barf Bowl?

____

____

____

____

____

Q12 What did you like least about Barf Bowl?

____

____

____

____

____

End of Block: LCAS

Start of Block: Science identity

Q5 To what extent do you agree or disagree with the following statements:

	Strongly disagree (1)	Somewhat disagree (2)	Somewhat agree (3)	Strongly agree (4)
I have a strong sense of belonging to the community of scientists (1)	○	○	○	○
I derive great personal satisfaction from working on a team that is doing important research (2)	○	○	○	○
I have come to think of myself as a 'scientist' (3)	○	○	○	○
I feel like I belong in the field of science (4)	○	○	○	○
The daily work of a scientist is appealing to me (5)	○	○	○	○

Q6 To what extent to do following statements sound like you?

	Not at all like me (1)	Somewhat not like me (2)	Somewhat like me (3)	Very much like me (4)
A person who thinks it is valuable to conduct research that builds the world's scientific knowledge (1)	○	○	○	○
A person who feels discovering something new in the sciences is thrilling (2)	○	○	○	○
A person who thinks discussing new theories and ideas between scientists is important (3)	○	○	○	○
A person who thinks that scientific research can solve many of today's world challenges (4)	○	○	○	○

End of Block: Science identity

Start of Block: GSE

Q1 From your experience this current academic semester, to what extent do you agree or disagree with the following statements:

	Strongly disagree (1)	Somewhat disagree (2)	Somewhat agree (3)	Strongly agree (4)
I can always manage to solve difficult problems if I try hard enough. (1)	o	o	o	o
If someone opposes me, I can find the means and ways to get what I want. (2)	o	o	o	o
It is easy for me to stick to my aims and accomplish my goals. (3)	o	o	o	o
I am confident that I could deal efficiently with unexpected events. (4)	o	o	o	o
Thanks to my resourcefulness, I know how to handle unforeseen situations. (5)	o	o	o	o
I can solve most problems if I invest the necessary effort. (6)	o	o	o	o
I can remain calm when facing difficulties because I can rely on my coping abilities. (7)	o	o	o	o

When I am confronted with a problem, I can usually find several solutions. (8)	○	○	○	○
If I am in trouble, I can usually think of a solution. (9)	○	○	○	○
I can usually handle whatever comes my way. (10)	○	○	○	○

End of Block: GSE

Start of Block: MW

Q7 Please select the option that best describes your experience of each over the last 2 weeks

	None of the time (1)	Rarely (2)	Some of the time (3)	Often (4)	All the time (5)
I've been feeling optimistic about the future (1)	○	○	○	○	○
I've been feeling useful (2)	○	○	○	○	○
I've been feeling relaxed (3)	○	○	○	○	○
I've been feeling interested in other people (4)	○	○	○	○	○
I've had energy to spare (5)	○	○	○	○	○
I've been dealing with problems well (6)	○	○	○	○	○
I've been thinking clearly (7)	○	○	○	○	○
I've been feeling good about myself (8)	○	○	○	○	○
I've been feeling close to other people (9)	○	○	○	○	○
I've been feeling confident (10)	○	○	○	○	○

I’ve been able to make up my own mind about things (11)	○	○	○	○	○
I’ve been feeling loved (12)	○	○	○	○	○
I’ve been interested in new things (13)	○	○	○	○	○
I’ve been feeling cheerful (14)	○	○	○	○	○

End of Block: MW
